# IgA response and protection following nasal vaccination of chickens with Newcastle disease virus DNA vaccine nanoencapsulated with Ag@SiO_2_ hollow nanoparticles

**DOI:** 10.1038/srep25720

**Published:** 2016-05-12

**Authors:** Kai Zhao, Guangyu Rong, Yan Hao, Lu Yu, Hong Kang, Xin Wang, Xiaohua Wang, Zheng Jin, Zhiyu Ren, Zejun Li

**Affiliations:** 1 School of Biological Science and Technology, University of Jinan, Jinan 250022, P. R.China; 2 Key Laboratory of Microbiology, School of Life Science, Heilongjiang University, Harbin 150080, P. R. China; 3 Key Laboratory of Functional Inorganic Material Chemistry, Ministry of Education, Laboratory of Physical Chemistry, Key Laboratory of Chemical Engineering Process & Technology for High-efficiency Conversion, School of Chemistry and Materials Science, Heilongjiang University, Harbin 150080, P. R. China; 4 Department of Avian Infectious Disease, Shanghai Veterinary Research Institute, Chinese Academy of Agricultural Sciences, Shanghai 200241, P. R. China

## Abstract

Newcastle disease caused by ND virus (NDV) is a highly contagious disease of birds. Vaccine for effective protection of poultry animals from NDV infection is urgently needed. Mucosal immunity plays a very important role in the antiviral immune response. In this study, a NDV F gene-containing DNA vaccine encapsulated in Ag@SiO_2_ hollow nanoparticles (pFDNA-Ag@SiO_2_-NPs) with an average diameter of 500 nm were prepared to assess the mucosal immune response. These nanoparticles exhibited low cytotoxicity and did not destroy the bioactivity of plasmid DNA, which could be expressed *in vitro*. The plasmid DNA was sustainably released after an initial burst release. *In vivo* immunization showed that the intranasal immunization of chickens with pFDNA-Ag@SiO_2_-NPs induced high titers of serum antibody, significantly promoted lymphocyte proliferation and induced higher expression levels of IL-2 and IFN-γ in a dose-dependent manner. These results indicated that the Ag@SiO_2_ hollow nanoparticles could serve as an efficient and safe delivery carrier for NDV DNA vaccine to induce mucosal immunity. This study has provided promising results for the further development of mucosal vaccines encapsulated in inorganic nanoparticles.

Newcastle disease (ND), a highly contagious disease of birds, affects many types of domestic and wild avian species. Its high susceptibility and mortality often cause epidemics in poultry populations^1^. The causative agent of this infectious disease is the virulent ND virus (vNDV), a member of genus *Avulavirus* in the subfamily *Paramyxovirinae* within the family *Paramyxoviridae*^2^. Two main viral genes, hemagglutinin-neuraminidase (HN) and fusion (F) genes, encode the two main HN protein and F protein. HN protein is responsible for viral attachment to specific receptors on the surface of the host cells while the F protein is an indispensable glycoprotein that enables the virus to bind and enter the host cells^3^,^4^,^5^. Nowadays, the vaccine inoculation still remains the main method to prevent ND. Current vaccination program for ND uses either attenuated vaccines or inactivated vaccines to induce protective immunity^6^. DNA vaccination can be a simple and promising tool for the development of new anti-NDV vaccines. Increasing attention has been focused on DNA vaccines because of their easy preparation and superior stability in ambient temperature and their ability to generate antigen-specific immune responses^7^,^8^. However, a significant obstacle to the successful development and applications of DNA vaccines has been the low efficacy of inducting immune response^9^. A few studies have shown that DNA vaccines usually administered via intramuscular injection can fail to reach the antigen-presenting cells (APCs) and therefore fail to induce immune responses because of the difficulty for them to pass through cell membranes^10^,^11^,^12^. Sun *et al*. reported that effective immunization of large animals required large amounts of DNA^13^, while reducing the DNA content was important because DNA-based vaccines can induce long-term both cellular and humoral immune responses in animals and humans^14^,^15^. It was recently suggested several measures, including optimization of plasmid DNA, improvement of delivery methods and their specificity for targeting the APCs, and the use of immunologic adjuvant, could increase the efficacy of DNA vaccines^16^,^17^.

Recent studies have indicated that the disadvantages of DNA vaccines could be avoided when the nanoparticle (NPs) mucosa immunity delivery system is built with nanomaterials. Because NPs are biodegradable and biocompatible, they cause lower cytotoxicity and can protect the antigen or DNA from being damaged under unfavorable conditions after systemic or mucosal administration^18^,^19^, and the uptake of NPs by APCs can be increased to facilitate the induction of potent immune responses^20^. Currently, many plasmid DNA delivery carriers and adjuvants, including liposome^21^, natural polymer^22^,^23^ and inorganic nanoparticles^24^,^25^,^26^, have been available. NPs such as polylactides and other polymers as DNA delivery carrier have been shown to improve the efficacy of DNA vaccines, and have attracted the increasing attention for potentially being used as the delivery carrier for a wide range of vaccines^27^.

Hollow spheres have a great potential for promising applications because of their lower effective density, higher specific surface area and many other advantageous properties^28^. In particularly, silver nanoparticles (AgNPs), with a broad spectrum of antibacterial activity against both gram-negative and gram-positive microorganisms, have become the focus of interest because they can be potentially applied in biological systems and medicine^29^,^30^,^31^. Recently, AgNPs have received considerable attentions as vaccine delivery system for their properties of lower cytotoxicity and full protection of the loaded plasmid DNA^32^. In addition, silica has also been widely used in biomedical applications, because of its merits of higher stability with respect to the changes in pH and concentration and easy functionalization^33^. Previous study had emphasized that hollow mesoporous silica nanoparticles could be used as vaccine carriers to improve both cellular and humoral immune responses^34^. Furthermore, unlike the simple nanoparticles, the core-shell nanoparticles have a core made of a material whose surface is coated with another material. This structural modification may improve their properties. For instance, it has been claimed that when they are applied in biological system, these core-shell nanoparticles appear to exhibit several major advantages over those of the simple nanoparticles, including lower cytotoxicity, higher dispersibility, bio- and cyto-compatibility, better conjugation with other bioactive molecules and increased thermal and chemical stability. Additionally, it has also been reported that the application of silica can significantly improve the thermostability and immunogenicity of viral vaccine^35^,^36^. The core/shell nanoparticles are mainly designed for biomedical applications based on their surface chemistry, which increases their affinity to drugs, receptors, and ligands etc.^37^. In the present study, we aimed to synthesize the Ag@SiO_2_ hollow nanoparticles with polystyrene (PS) microspheres as templates for preparing hollow spheres. We reported the successful preparation of the Ag@SiO_2_ hollow nanoparticles. Moreover, we demonstrated that these Ag@SiO_2_ hollow nanoparticles also possessed the properties of uniform structure, lower cytotoxicity, higher stability, full protection of the loaded plasmid DNA and controllable synthesis as compared with those of polymeric nanoparticles prepared in our previous study^38^,^39^. The Ag@SiO_2_ hollow nanoparticles prepared in this study were used as the delivery carrier of NDV DNA vaccine with plasmid DNA containing the F gene designated as pFDNA-Ag@SiO_2_-NPs. Their characteristics as a delivery carrier for NDV DNA vaccine were studied and their abilities to induce immune responses and to protect specific pathogen free (SPF) chickens from being infected by NDV after intranasal administration were also assessed.

## Results

### Characterization of the Ag@SiO_2_ hollow nanoparticles

**Figure 1:**
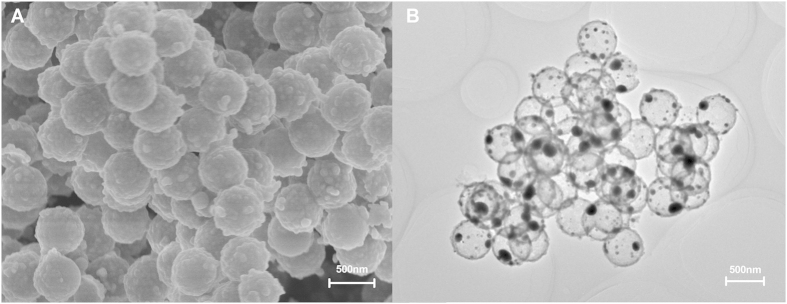
Electron microscopy micrograph of the Ag@SiO_2_ hollow nanoparticles. (**A**) Shows scanning electron microscopy micrograph; and (**B**) shows transmission electron microscopy micrograph.

For the synthesis of Ag@SiO_2_ hollow nanoparticles, monodisperse PS microspheres (Fig. S1A) were synthesized through an emulsion polymerization in a water-ethanol system for the use as the templates for subsequent Ag deposition. We observed that the Ag covered the surface of PS microspheres (Fig. S1B). The Ag@SiO_2_ hollow nanoparticles prepared herein were spherical in shape with the size of 490–500 nm, as shown in Fig. 1. The N_2_ adsorption–desorption isotherms and pore size distribution plots of Ag@SiO_2_ hollow nanoparticles were shown in Fig. S2. The Ag@SiO_2_ hollow nanoparticles retained type IV Brunauer Emmett and Teller (BET) isotherms (Fig. S2A), corresponding to a type H3 pile pore structure. The corresponding BJH pore size distribution plots showed that Ag@SiO_2_ hollow nanoparticles had the BET surface area of 20.4 m^2^/g and a pore size of 110 nm (Fig. S2B). In this case, Ag@SiO_2_ hollow nanoparticles displayed hollow architecture. Their pore surface and larger surface area enable them to contact with more DNA plasmid and to achieve sustained release.

### Preparation of the pFDNA-Ag@SiO_2_-NPs

**Figure 2:**
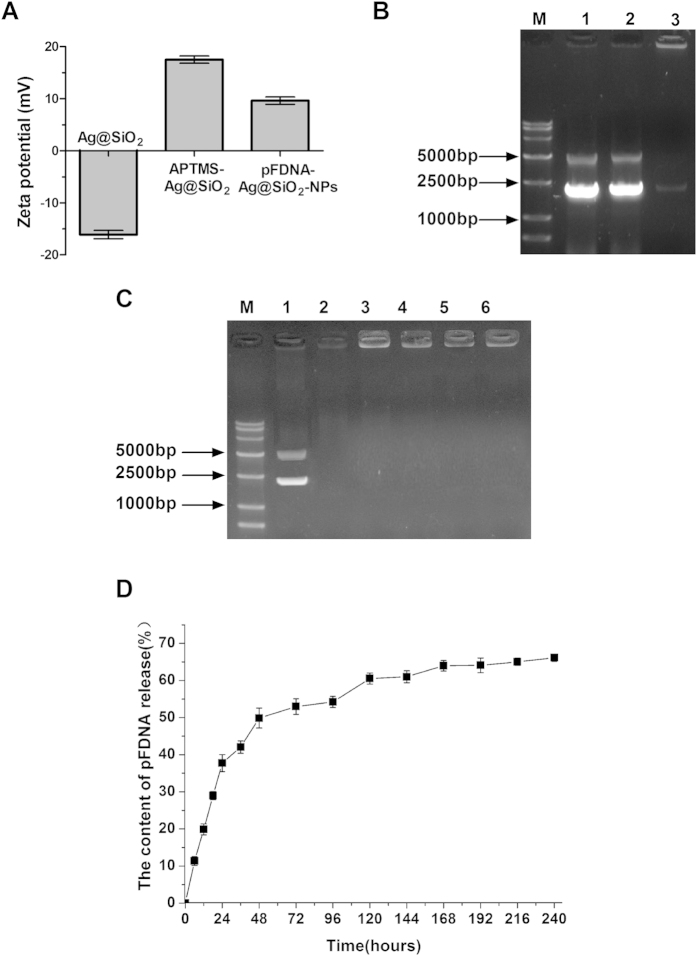
(**A**) Zeta potential of Ag@SiO_2_ hollow nanoparticles, APTMS-Ag@SiO_2_ hollow nanoparticles and pFDNA-Ag@SiO_2_-NPs. Data are presented as the mean ± standard deviation (n = 5). (**B**) The combination of the plasmid pVAX I-F (o) DNA with Ag@SiO_2_ hollow nanoparticles, APTMS-Ag@SiO_2_ hollow nanoparticles. Lane 1 shows the plasmid DNA; Lane 2 shows the Ag@SiO_2_ hollow nanoparticles mixed with plasmid DNA; Lane 3 shows the APTMS-Ag@SiO_2_ hollow nanoparticles loaded the plasmid DNA (pFDNA-Ag@SiO_2_-NPs); M: DNA Marker DL 15000. (**C**) Detection of APTMS-Ag@SiO_2_ hollow nanoparticles loaded DNA about protection and delivery property. M: DNA marker DL 15000; Lane 1: the plasmid pVAX I-F (o) DNA; Lane 2: the plasmid pVAX I-F (o) DNA treated by DNase I for 30 min; Lanes 3-6: pFDNA-Ag@SiO_2_-NPs treated by DNase I for 30, 60, 120, and 180 min, respectively; and (**D**) Release behavior of pFDNA-Ag@SiO_2_-NPs in the PBS solution (pH = 7.4) at 37 °C. Data are presented as the mean ± standard deviation (n = 5).

Ag@SiO_2_ hollow nanoparticles were combined with the plasmid pVAX I-F (o) DNA mainly via electrostatic interaction. The zeta potentials of Ag@SiO_2_ hollow nanoparticles, APTMS-Ag@SiO_2_ hollow nanoparticles and pFDNA-Ag@SiO_2_-NPs were −16.1 mV, +17.5 mV and +9.62 mV, respectively (Fig. 2A). As shown in Fig. 2B, the plasmid pVAX I-F (o) DNA was adsorbed on Ag@SiO_2_ hollow nanoparticles modified by amidogen and stranded in spotting holes (Lane 3). The encapsulation efficiency (EE) of pFDNA-Ag@SiO_2_-NPs was 70.96 ± 5.74% (n = 5). These results indicated the feasibility of using the electrostatic adsorption method for preparation of pFDNA-Ag@SiO_2_-NPs via the interaction between Ag@SiO_2_ hollow nanoparticles and the plasmid pVAX I-F (o) DNA.

### DNA protection, *in vitro* release and *in vitro* expression of the pFDNA-Ag@SiO_2_-NPs

**Figure 3:**
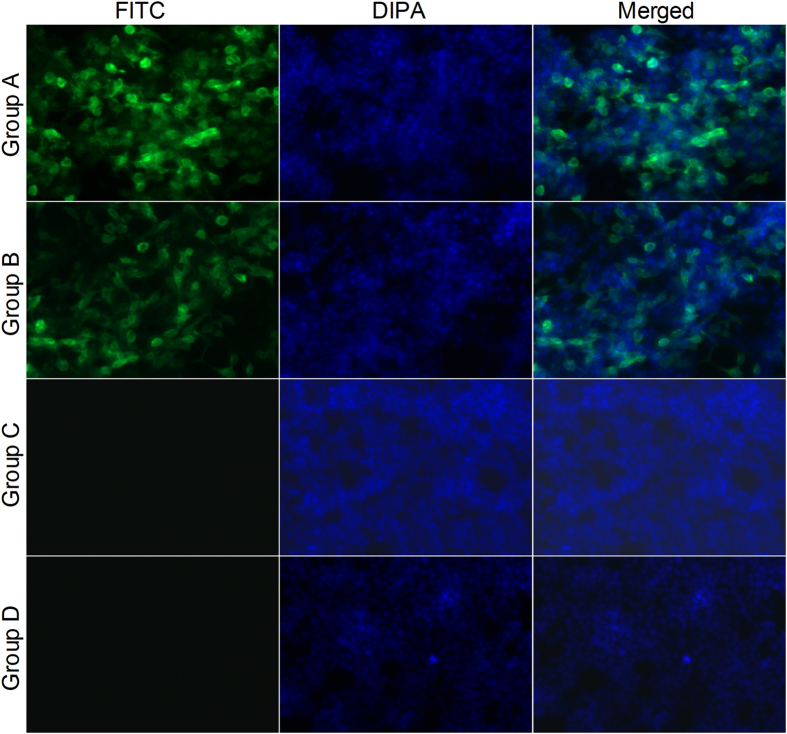
*In vitro* expression of the pFDNA-Ag@SiO_2_-NPs in 293 T cells detected by the indirect immunofluorescence analysis (×20). Group A: pFDNA-Ag@SiO_2_-NPs; Group B: pFDNA-Ag@SiO_2_-NPs treated by DNase I; Group C: Blank Ag@SiO_2_ hollow nanoparticles; Group D: 293 T cells as the negative control.

To determine whether the Ag@SiO_2_ hollow nanoparticles can prevent DNA from degradation by endonuclease (DNase), the plasmid pVAX Ι-F (o) DNA and DNA-Ag@SiO_2_ compound were firstly treated with DNase Ι and the enzymatically digested products were then analyzed with agarose gel electrophoresis, respectively. The plasmid pVAX Ι-F (o) DNA was degraded by incubation with DNase Ι within 30 min (Lane 2, Fig. 2C) whereas the plasmid pVAX Ι-F (o) DNA encapsulated in APTMS-Ag@SiO_2_ nanoparticles was protected from degradation by DNase Ι (Lanes 3-6, Fig. 2C). To further test the stability of pFDNA-Ag@SiO_2_-NPs after being treated with DNase I, the entire F genes of NDV were amplified by PCR. The PCR products were sequenced and analyzed by agarose gel electrophoresis (Fig. S3), but no mutation was found. These results demonstrated that APTMS-Ag@SiO_2_ encapsulation protected the DNA from DNase I digestion. The *in vitro* release of the plasmid DNA results suggested that the APTMS-Ag@SiO_2_ hollow nanoparticles could be used as a potential non-viral gene delivery system (Fig. 2D). As shown in Fig. 3, both Group A that was transfected with pFDNA-Ag@SiO_2_-NPs but without being treated with DNase I and Group B of pFDNA-Ag@SiO_2_-NPs treated with DNase I induced specific fluorescence. However, the blank Ag@SiO_2_ (Group C) and the negative cell control (Group D) exhibited no observable fluorescence. Furthermore, the expression of pFDNA-Ag@SiO_2_-NPs in 293 T cells could be clearly observed, further demonstrating that Ag@SiO_2_ hollow nanoparticles can protect the DNA from DNase I digestion.

### Biological safety of the pFDNA-Ag@SiO_2_-NPs

To determine the biological safety of the pFDNA-Ag@SiO_2_-NPs, the survival rates of chicken embryo fibroblast (CEF) cells were measured in the presence of different nanoparticles at different concentrations. The survival rate of CEF cells were over 77% after being incubated with Ag@SiO_2_ hollow nanoparticles at different concentrations (Fig. S4), and no significant changes in cell morphology were observed as compared to those of the control cells (*P* > 0.05). These results indicated that the Ag@SiO_2_ hollow nanoparticles and pFDNA-Ag@SiO_2_-NPs caused little cytotoxicity but had a higher level of biological safety. The feed intake, water intake, and mental state of SPF chickens immunized i.m. with pFDNA-Ag@SiO_2_-NPs and immunized i.n. with pFDNA-Ag@SiO_2_-NPs were all normal as compared with those of the control group. No pathological changes were observed in the immunized chickens. Thus, both the morbidity and mortality were 0%. These results revealed that the vaccination of chickens with the pFDNA-Ag@SiO_2_-NPs mediated via the administration routes tested was safe.

### Assay of serum hemagglutination inhibition (HI) antibody

**Figure 4:**
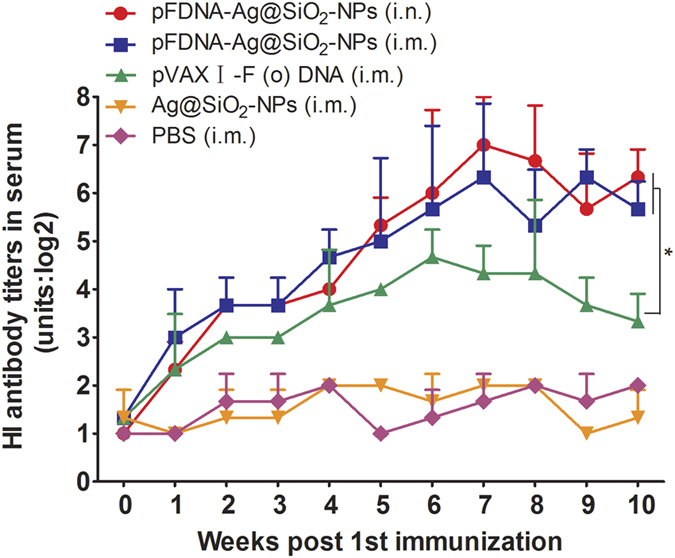
HI antibody titer in serum of SPF chickens immunized with the PBS i.m., blank Ag@SiO_2_-NPs i.m., plasmid pVAX I-F (o) DNA i.m., pFDNA-Ag@SiO_2_-NPs i.m., and pFDNA-Ag@SiO_2_-NPs i.n. Data are presented as the mean ± standard deviation (n = 5). “*” indicates statistically significant difference at *P* < 0.05.

As shown in Fig. 4, the antibody titers were not changed significantly during the first 1–10 weeks post the immunization in chickens being immunized with PBS or blank Ag@SiO_2_-NPs. Conversely, the antibody titers of chickens being immunized with the pFDNA-Ag@SiO_2_-NPs i.m. and i.n. were quickly increased at the third week and peaked at the seventh week post the immunization. In addition, the difference in antibody title between two immunization routes was not statistically significant (*P* > 0.05). The antibody titles in chickens immunized with the pFDNA-Ag@SiO_2_-NPs via two immunization routes were significantly higher than those of chickens immunized with the plasmid pVAX I-F (o) DNA i.m. (*P* < 0.05), suggesting that the pFDNA-Ag@SiO_2_-NPs are able to stimulate the immune responses of chickens and to maintain them at high levels for a long time.

### Assay of IgA antibody

**Figure 5:**
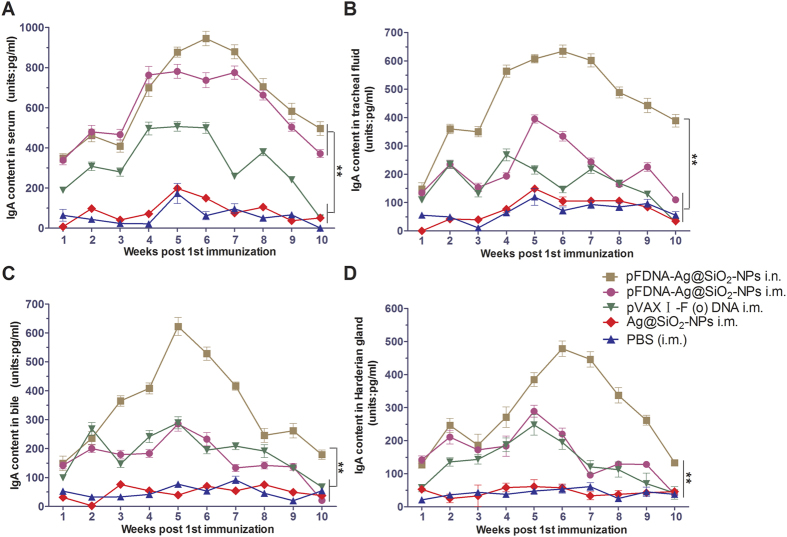
IgA antibody titers in serum (**A**), tracheal fluid (**B**), bile (**C**), and Harderian gland (**D**) of SPF chickens immunized with the PBS i.m., blank Ag@SiO_2_-NPs i.m., plasmid pVAX I-F (o) DNA i.m., pFDNA-Ag@SiO_2_-NPs i.m., and pFDNA-Ag@SiO_2_-NPs i.n. IgA antibody titers in these samples were detected with ELISA. Data are presented as the mean ± standard deviation (n = 3). “**” indicates statistically significant difference at *P* < 0.01.

The changes in IgA contents in serum, tracheal fluid, bile, and Harderian gland before and after the chickens were immunized with the pFDNA-Ag@SiO_2_-NPs were assayed and the results were shown in Fig. 5. The titers of IgA antibody in chickens immunized with pFDNA-Ag@SiO_2_-NPs i.n. were significantly higher (*P* < 0.01) and the periods of IgA antibody secretion in their tracheal fluid (Fig. 5B), bile (Fig. 5C), and Harderian gland (Fig. 5D) were also longer than those of chickens in other groups. While the titers of IgA antibody in serum between two groups with pFDNA-Ag@SiO_2_-NPs immunization were not significantly different, they were significantly higher than those in the plasmid pVAX I-F (o) DNA and the control group (*P* < 0.01, Fig. 5A).

### Assay of lymphocyte proliferation and analysis of cytokine responses

**Figure 6:**
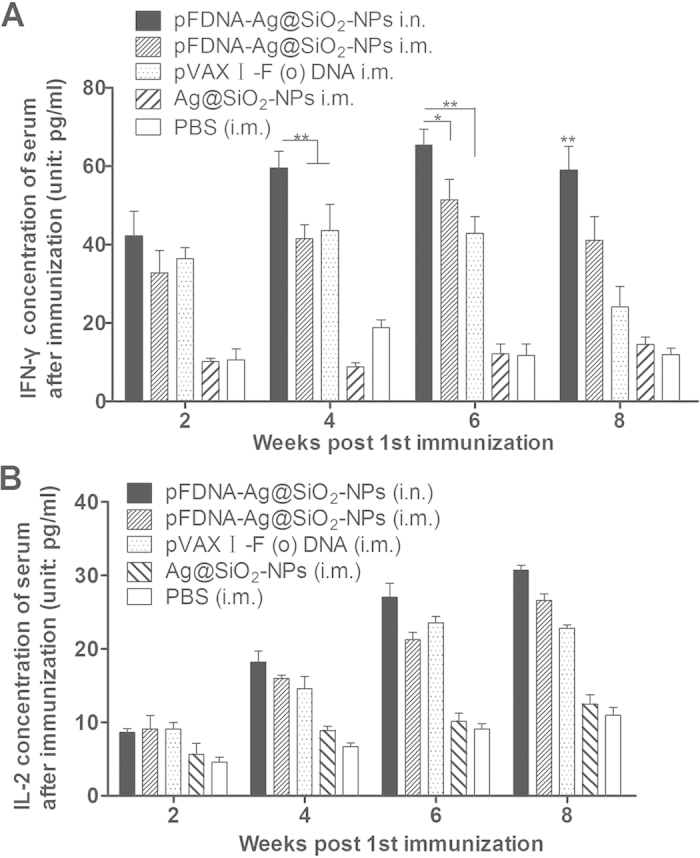
Contents of IFN-γ (**A**) and IL-2 (**B**) of SPF chickens immunized with the PBS i.m., blank Ag@SiO_2_-NPs i.m., plasmid pVAX I-F (o) DNA i.m., pFDNA-Ag@SiO_2_-NPs i.m., and pFDNA-Ag@SiO_2_-NPs i.n. Data are presented as the mean ± standard deviation (n = 3). “*” indicate statistically significant difference at *P* < 0.05; “**” indicate statistically significant difference at *P* < 0.01.

The expression levels of inflammatory cytokines have been used as one of the stimulation indices (SIs) for immunization since they play a central role in the modulation of immune responses. In this study, we detected the levels of two major inflammatory cytokines, IFN-γ and IL-2, in the serum samples of chickens in each experimental group by enzyme-linked immunosorbent assay (ELISA). As shown in Fig. 6, the level of IFN-γ (Fig. 6A) was significantly higher in chicken immunized with pFDNA-Ag@SiO_2_-NPs i.n. than those of chickens in other groups (*P* < 0.01), However, no statistically significant differences in the levels of IL-2 (Fig. 6B) were seen among the chickens in pFDNA-Ag@SiO_2_-NPs i.n., pFDNA-Ag@SiO_2_-NPs i.m. and pVAX I-F (o) DNA i.m. groups (*P* > 0.05). These results suggested that pFDNA-Ag@SiO_2_-NPs i.n. could facilitate the cellular immune responses by enhancing the secretion of inflammatory cytokine IFN-γ.

### Protective efficacy of the pFDNA-Ag@SiO_2_-NPs

No clinical symptoms, mortality and histopathological changes were observed in chickens immunized with the pFDNA-Ag@SiO_2_-NPs i.n. and followed by being challenged with the highly virulent NDV strain F_48_E_9_. Thus, the protective efficacy was 100% (Table 1). The protective efficacy was 80% for the chickens immunized with the pFDNA-Ag@SiO_2_-NPs i.m. and 70% for the chickens immunized with plasmid pVAX I-F (o) DNA i.m, respectively. However, all the chickens immunized with PBS or with blank Ag@SiO_2_ and challenged with NDV strain F_48_E_9_ were dead in 2–5 d and thus, the protective efficacy was 0%. Moreover, all the dead chickens from the chickens in the groups of pFDNA- Ag@SiO_2_-NPs i.m., the plasmid pVAX I-F (o) DNA i.m., blank Ag@SiO_2_-NPs i.m. and PBS i.m. displayed the typical pathological changes of ND, including mucosal hemorrhages in proventriculus papillae, fatty heart, duodenum and the whole intestines. These results showed that the pFDNA-Ag@SiO_2_-NPs being administrated via i.n. route quickly induced the effective mucosal immune response.

## Discussions

NDV infection is still one of the important infectious diseases in poultry animals with serious long-term morbidities and mortalities. Thus, the effective strategies designed for anti-NDV are urgently needed. DNA vaccines for NDV can offer an attractive option against NDV infection, and have drawn increasing attention for a long time. As a new type of vaccines, DNA vaccines possess several advantages over the classical antigen vaccines. They are capable of eliciting both humoral and cellular immune responses^40^. However, DNA vaccines are easily degraded *in vivo* by endogenous nucleases and thus, their bioavailability is not high and long enough. Therefore, a safer and more efficient delivery system that not only is able to protect DNA vaccines from degradation by endogenous nucleases but also is able to increase and prolong their bioavailability is certainly needed.

Silver nanoparticles as a delivery carrier cause lower cytotoxicity and can provide full protection for the loaded plasmid DNA^32^, while hollow mesoporous silica nanoparticles as a vaccine carrier can improve both cellular and humoral immune responses^34^. Furthermore, core-shell nanoparticles also have numerous merits, including the lower cytotoxicity, the increased dispersibility, bio- and cyto-compatibility, and better conjugation with other bioactive molecules etc.^37^. Hence, in the present study, the Ag@SiO_2_ hollow nanoparticles were synthesized and used as the delivery carrier for NDV DNA vaccine. With the Ag@SiO_2_ hollow nanoparticles, the sustained release of veterinary antigen genes could be realized and the desired mucosal immunity could be effectively induced. It has been known that the amino acid sequence surrounding the fusion (F) protein cleavage site is mainly responsible for virulence of NDV^41^. In this study, we focused on preparation of F gene-containing plasmid DNA of NDV encapsulated in the Ag@SiO_2_ hollow nanoparticles using an electrostatic adsorption method. Zeta potential of the Ag@SiO_2_ hollow nanoparticles was −16.10 mV, which was changed to 17.50 mV after being modified with 3-aminopropyltrimethoxysilane (APTMS) (Fig. 2A). The combination of the negatively charged DNA plasmids with the positively charged Ag@SiO_2_ NPs being modified by APTMS could enhance both the bio-adhesivity and the site-specific applications in the controlled delivery systems^38^,^42^. The average diameter of Ag@SiO_2_ hollow nanoparticles was about 500 nm (Fig. 1). Nanoparticle size is an important factor determining whether the nanoparticles can pass through the mucosal surfaces. It was reported that the nanoparticles could more easily pass through the mucosal barrier^43^ and could improve gene transfection efficiency^44^ when their size was smaller than 1 μm. This indicated that the small sized-nanoparticle carriers could help to increase antigen contact area with the mucous membranes and improve the mucosal uptake, thereby enhancing the bioavailability of antigen and stimulating the body to produce an effective immune response. These important advantages of Ag@SiO_2_ hollow nanoparticles could be attributed to their pore surface and larger surface area, which make Ag@SiO_2_ an ideal carrier for the delivery of large amount of DNA vaccine. We have determined the EE for incorporating DNA into nanoparticles, because it determines the effectiveness of the gene delivery and subsequent expression of the inserted genes encoding antigens both *in vitro* and *in vivo*^45^. The EE was 70.96 ± 5.74% in our study. The Ag@SiO_2_ hollow nanoparticles could fully protect DNA from digestion by DNase and deliver the plasmid DNA into 293 T cells (Figs 2 and 3). On the other hand, the safety of the pFDNA-Ag@SiO_2_-NPs was tested by *in vitro* cytotoxicity analysis and their biological safety was also tested in chickens before immunizations. All the results demonstrated that the procedures of preparation and vaccination of these nanoparticle-carried DNA vaccines were safe, and that the immunogenicity of the plasmid DNA was retained after being encapsulated into these nanoparticles for the production of the vaccines. Efficient and sustained release of vaccine can stimulate the body to produce antibodies and to extend the protective term of vaccine. In the present study, the *in vitro* release assay showed that the release process of the plasmid DNA included both a rapid release phase and a stable and steady release phase (Fig. 2D). Internalization of Ag@SiO_2_ hollow nanoparticles into the acidic environments (e.g. endosome and lysosome) can lead to the collapse of material surface and the sustained release of vaccine.

In birds, their peripheral lymphoid organs include Harderian glands, spleen and all the mucosa-associated lymphoid tissues (MALTs), including the respiratory, urinary and alimentary tracts^46^. Lymphocytes are the important constituents of immune system and approximately 50% of lymphocytes are present in the MALTs, which are located along the surfaces of all the mucosal tissues. B-cells, CD4^+^ and CD8^+^ T-cells, antigen-presenting dendritic cells (DCs), macrophages, occasionally mast cells and eosinophils in the interfollicular region constitute MALTs. Thus, MALTs contain all the essential cell types to instigate an immune response^47^. Mucosal immune system includes both humoral and cellular immunities, which were reported to play important roles in the host’s defense against NDV infection^48^. For many avian infectious diseases, cellular immune response was known to play a dominant role in immune protection^49^.

IgA plays an important role at the effecter arm of the mucosal system while both IgG and IgM are actively produced at MALTs and transported across mucosal cells, thereby increasing their concentrations there. Detection of IgG at mucosal site can be resulted from the passive leakage across the mucosal surface. IgA is considered as the primary mucosal antibody and, along with IgM, it can be transported across the epithelial barrier, which provides potent immunity against a large number of viral infections^50^. The Harderian gland of chicken, a part of the eye-associated lymphoid tissues, is located in the orbit behind the eye where it also plays an important role in the adaptive mucosal immune response upon ocular exposure to avian pathogens. In this research, we not only detected the IgA concentrations in serum, bile, and trachea, but also detected its concentration in Harderian gland. Interestingly, the results of vaccination of SPF chickens showed that the levels of both HI antibody and IgA of chickens given intranasal immunization with pFDNA-Ag@SiO_2_-NPs were higher than those of chickens given intramuscular injection and intranasal immunization with the naked plasmid DNA (Figs 4 and 5). It was reported that the effective immunization strategies for protection against infection of influenza virus involved the induction of mucosal immune responses at the nasal mucosal epithelium^51^,^52^. Increased muco adhesion through the use of nanoparticles has the benefit of enabling the vaccine to gain a better access to the lymphoid tissue^53^ and to improve the production of IgG^54^ by increasing its amount and prolonging the time of its interaction with the mucosal cells. It has been known that some nanoparticles are capable of opening the tight junctions and enhancing delivery^55^,^56^. We believed that the Ag@SiO_2_ hollow nanoparticles might also have this feature. With the differentiation of more B cells into IgA plasma cells, more IgA antibody is produced. In summary, intranasal immunization of chickens with pFDNA-Ag@SiO_2_-NPs not only increased the local levels of both IgA and HI antibodies, but also caused a systemic immune response.

Inflammatory cytokines play pivotal roles as natural mediators and regulators of the immune response^57^. In chickens, the responses of Th1-type cytokines (IFN-γ, IL-2, and IL-12) predominate in response to infections of intracellular pathogens and generally work to augment cellular immunity^58^. Since cytokines play a critical role in development of cellular immunity and prevention of viral infections^59^, the production levels of two important cytokines, IFN-γ and IL-2, an indication of a Th1-type response, were measured. Higher levels of both IFN-γ and IL-2 were detected in the groups of chickens vaccinated with the pFDNA-Ag@SiO_2_-NPs (Fig. 6). Interestingly, more Th1-type cytokines were secreted in chickens intranasally immunized with pFDNA-Ag@SiO_2_-NPs than those in other groups, indicating that the constructed DNA vaccines against NDV induce Th1-type cellular responses. Ag@SiO_2_ was shown to enhance cellular immune responses and the production of cytokines for NDV DNA vaccination. In our research, Ag@SiO_2_ hollow nanoparticles displayed many advantages as a carrier for DNA vaccines. They are not only capable of delivering DNA into 293 T cells, but also are capable of enhancing mucosal immune responses. Our results clearly demonstrated that the Ag@SiO_2_ hollow nanoparticles could be used as a delivery vehicle for NDV DNA vaccine containing the F gene plasmid DNA. Intranasal immunization of pFDNA-Ag@SiO_2_-NPs induced stronger humoral, cellular and mucosal immunities and achieved sustained release of vaccine. Future studies may yield more promising results in the use of nanoparticles as carriers of DNA vaccine.

## Materials and Methods

### Ethics statement

Care of laboratory animals and experimentation on animals were done in accordance with animal ethics guidelines and approved protocols. All the animal studies were approved by the Animal Ethics Committee of the Harbin Veterinary Research Institute of the Chinese Academy of Agricultural Sciences (CAAS), China (SCXK (H) 2013-001).

### Synthesis of Ag@SiO_2_ hollow nanoparticles

Well-dispersed and uniform Ag@SiO_2_ hollow nanoparticles were fabricated via a template method using polystyrene (PS) as templates, followed by a subsequent heat treatment^60^,^61^,^62^. Briefly, preparation of Ag@SiO_2_ hollow nanoparticles included the following procedures: (1) PS microspheres were firstly synthesized by emulsion polymerization in a water-ethanol system and then modified by polyethylenimine (PEI) (0.98 g/ml) (Sigma-Aldrich, St. Louis, MS, USA); (2) PS microspheres were firstly modified with PEI and then added into 100 ml of aqueous solution containing 0.51 g of AgNO_3_ (Sinopharm Chemical Reagent, Beijing, China). The solution was heated at 100 °C for 1 h, and then 0.25 g of sodium citrate (Sinopharm Chemical Reagent) was added after the solution cooled to room temperature. PS@Ag composite nanoparticles were obtained; (3) PS@Ags were modified by polyvinylpyrrolidone (PVP) (Sigma-Aldrich). 0.17 g of PS@Ags modified with PVP was added to a mixture containing 25 ml of ethanol, 1.5 ml of H_2_O, and 0.5 ml of tetraethyl orthosilicate (TEOS) (Tianjin Kermel Chemical Reagent, Tianjin, China). The mixture was slowly heated up to 50 °C in an oil bath and 25 ml of ammonia was added quickly, and the reaction was terminated at the 25^th^ min after reaction began. PS@Ag@SiO_2_ core/shell composite nanoparticles were collected through centrifugation and washed with ethanol 3 times. The double-shell Ag@SiO_2_ hollow nanoparticles were obtained by dissolving PS core with calcination at 400 °C for 1 h, and then heated to 500 °C for 3 h. The morphological characteristics and Zeta potentials of the Ag@SiO_2_ hollow nanoparticles were examined by scanning electron microscopy (SEM)(Hitachi S-4800, Hitachi Ltd, Tokyo, Japan), transmission electron microscopy (TEM)(JEM-200EX, Hitachi Ltd, Tokyo, Japan) and Zeta Sizer 2000 (Malvern, UK).

### Preparation of the pFDNA-Ag@SiO_2_-NPs as vaccine

The eukaryotic expression plasmid pVAX I-optiF that carries and drives the expression of the F gene of NDV was provided by State Key Laboratory of Veterinary Biotechnology, Harbin Veterinary Research Institute, Chinese Academy of Agricultural Sciences (Harbin, Heilongjiang, China). This eukaryotic expression plasmid was firstly extracted by the alkaline lyses method^63^ and then encapsulated in Ag@SiO_2_ nanoparticles (pFDNA-Ag@SiO_2_-NPs) by an electrostatic adsorption method. The Ag@SiO_2_ nanoparticles were functionalized with terminal amine groups (-NH_2_) by 3-aminopropyltrimethoxysilane (APTMS, J&K, USA). 18 μl of the plasmid pVAX I-F (o) DNA (545 ng/μl) was added to 100 μl of the Ag@SiO_2_ hollow nanoparticles suspensions (1.5 mg/ml). The mixture was incubated at room temperature for 30 min. The nanoparticle size and Zeta potentials of the resulting pFDNA-Ag@SiO_2_-NPs were measured by a Zeta Sizer 2000 obtained from Malvern Instruments (Malvern, UK). Encapsulation efficiency (EE) was measured as follows^38^: EE (%) = (W_0_–W_1_)/W_0_ × 100%, where W_0_ is total amount of the plasmid DNA added and W_1_ is amount of the free plasmid DNA. All the measurements were performed five times.

### Dnase I protection, *in vitro* release assay and *in vitro* transfection assay

To test stability of the pFDNA-Ag@SiO_2_-NPs, the pFDNA-Ag@SiO_2_-NPs suspension (10 μl, equivalent to 3.0 μg of the naked plasmid DNA) were incubated with 50 units of DNase I (TaKaRa, Japan) at 37 °C in 50 μl of reaction buffer for 30, 60, 120 and 180 min, respectively. The reaction was terminated by adding 100 μl of termination solutions at 65 °C for 10 min. The naked plasmid pVAX I-F (o) DNA (3.0 μg/μl of 20% of Na_2_SO_4_) was incubated at 37 °C for 30 min and used as the negative controls. The suspension was analyzed by loading it onto 0.8% agarose gel and the electrophoresis was run with Tris-acetate buffer at 100 V for 30 min. The F genes were amplified using ordinary PCR assay. Sequences of the PCR-amplified products were analyzed using DNASTAR software. An *in vitro* release assay was carried out to determine the release of the plasmid pVAX I-F (o) DNA from the Ag@SiO_2_-NPs^39^. Briefly, the pFDNA-Ag@SiO_2_-NPs suspension was centrifugalized at 16, 000 r/min for 10 min at 4 °C, and then the precipitation was re-suspended with 1.5 ml of PBS (pH 7.4) and stirred at 100 r/min at 37 °C. The concentration of the released plasmid pVAX I-F (o) DNA in the supernatant was determined by using UV spectrophotometry. All the experiments were performed five times.

To verify the *in vitro* expression of plasmid pVAX I-F (o) DNA encapsulated in the Ag@SiO_2_-NPs into the NDV antigen, the *in vitro* transfection of these nanoparticles into 293-T cell grown in polylysine-treated 6-well plates at 37 °C in a CO_2_ (5%) incubator were analyzed when the cell growth reached 80% confluence and the expression of plasmid DNA in the transfected cells was monitored with an indirect immunofluorescent test. The NDV positive serum (Shanghai Veterinary Research Institute, SVRI, China) and fluorescein isothiocyanate-labeled goat-anti-chicken IgG (Sigma) were diluted at 1:1, 000 and 1:2, 000, respectively. Nuclei were stained with the 1 μg/ml of 4′, 6-diamidino-2-phenylindole (DAPI) for 5 min at room temperature. Epifluorescence images were acquired using an Axio observer Z1 microscope (Zeiss).

### *In vitro* cytotoxicity assay and *in vivo* biological safety assay

To test the safety of the Ag@SiO_2_-NPs as a DNA vaccine delivery carrier for mucosal immune, the *in vitro* cytotoxicity was evaluated by using CCK-8 reagent (Dojindo Ltd, Japan), and the survival rate of chicken embryonic fibroblast (CEF) cells was determined by measuring OD_450_. The dose-dependent *in vitro* cytotoxicity was detected with Ag@SiO_2_ nanoparticles and pFDNA-Ag@SiO_2_-NPs at the concentrations of 1, 10, 100 and 1000 μg/ml, respectively. For the *in vivo* biological safety assay, thirty 4-week-old SPF chickens from Harbin Pharmaceutical Group Bio-vaccine Co. Ltd were randomly grouped into three groups with 10 chickens in each group. Chickens in Group 1 were immunized intranasally (i.n.) with 0.2 ml of the pFDNA-Ag@SiO_2_-NPs containing a total of 200 μg of the plasmid pVAX I-F (o) DNA; Chickens in Group 2 were immunized intramuscularly (i.m.) with 0.2 ml of the naked plasmid pVAX I-F (o) DNA; and chickens in Group 3 were immunized i.m. with 0.2 ml of phosphate buffered saline (PBS, pH 7.4). Any abnormal changes in chickens were continuously observed and recorded for 21 d.

### Immunization of SPF chickens

30-day-old SPF chickens from Harbin Pharmaceutical Group Bio-vaccine Co. Ltd were randomly divided into five groups with 45 chickens in each group. Chickens in Group 1 were immunized i.m. with PBS as a negative control; chickens in Group 2 were immunized i.m. with the blank Ag@SiO_2_-NPs; chickens in Group 3 were immunized i.m. with 200 μg of the naked plasmid pVAX I-F (o) DNA (0.2 ml); chickens in Groups 4 and 5 were immunized with 0.2 ml of the pFDNA-Ag@SiO_2_-NPs containing 200 μg of naked pVAX I-F (o) plasmid DNA) i.m. and i.n., respectively. At 2 weeks after the first immunization, chickens in groups 1, 2, 3, 4 and 5 were boosted with PBS, the blank Ag@SiO_2_-NPs, the naked plasmid pVAX I-F (o) DNA and the pFDNA-Ag@SiO_2_-NPs the same dosages via the same routes as the first immunization, respectively. Care of laboratory animals and animal experimentation were performed in accordance with animal ethics guidelines with the protocols approved by Institutional Committee for Animal Care and Use.

### Detection assays of HI antibody and IgA antibody

Blood samples were collected from the wing veins of five chickens in each of the five groups at 7, 14, 21, 28, 35, 42, 49, 56, 63 and 70 d post the 1st immunization, and then the serum samples were separated by centrifugation at 3000 r/min for 10 min. The titer of the NDV-specific HI antibody in serum was detected by HI test (n = 5). To evaluate the mucosal immune response, serum, tracheal fluid, bile, and Harderian glands were collected from three chickens at 7, 14, 21, 28, 35, 42, 49, 56, 63 and 70 d post 1st immunization. Mucosal extracts were obtained by centrifugation to collect the supernatant. The titer of IgA antibody was detected by enzyme linked immunosorbent assay (ELISA)-sandwich technique according to the instruction manual of NDV IgA ELISA Kit (Rapidbio Co. Ltd., Beijing, China) (n = 3).

### ELISAs for cytokines

For cytokine assays, serum samples were collected from three chickens at 2, 4, 6, and 8 weeks post 1st immunization. The amounts of IFN-γ and IL-2 in the collected serum samples were determined using the IFN-γ ELISA Kit (Abcam Co. Ltd., Shanghai, China) and IL-2 ELISA Kit (Santa Cruz Co. Ltd., Santa Cruz, CA, USA), respectively. All the operations were performed according to the procedures described for the cytokine ELISA kits.

### Protection efficacy against the infection of NDV strain F_48_E_9_

An experiment was carried out to evaluate the ability of pFDNA-Ag@SiO_2_-NPs to protect chickens against the infection of NDV strain F_48_E_9_ (Harbin Pharmaceutical Group Bio-vaccine Co. Ltd.) after inoculation. The genotype of NDV strain F_48_E_9_, a highly virulent strain (MDT ≤ 60 h, ICPI > 1.6) was IX. When the levels of serum antibody for ND of the immunized chickens in pFDNA-Ag@SiO_2_-NPs i.n. group and pFDNA-Ag@SiO_2_-NPs i.m. group were increased to 6.0 log 2 at the seventh week after the first immunization, ten chickens were selected randomly from each of the five groups and were infected i.m. with 100 μl of the highly virulent NDV strain F_48_E_9_ at a viral titer of 10^4^ EID_50_/0.1 ml at the same time. Feed, water, mental state, clinical symptoms and mortality of these chickens were continuously observed and recorded for 35 d. The infected chickens and the corresponding negative control chickens were euthanized and their glandular stomach, duodenum and myocardium were collected for the examination by histological staining.

### Statistical analysis

All the experimental results were expressed as mean values ± standard deviation (SD). One-factor analysis of variance (ANOVA) was employed to evaluate the statistical differences among different groups with SPSS 19.0 software. The difference between groups with *P*-value of <0.05 and <0.01 was considered to be statistically significant.

## Additional Information

**How to cite this article**: Zhao, K. *et al*. IgA response and protection following nasal vaccination of chickens with Newcastle disease virus DNA vaccine nanoencapsulated with Ag@SiO_2_ hollow nanoparticles. *Sci. Rep*. **6**, 25720; doi: 10.1038/srep25720 (2016).

## Supplementary Material

Supplementary Information

## Figures and Tables

**Table 1:** Protective efficacy of the immunized SPF chickens after being challenged with the highly virulent NDV strain F_48_E_9_.

Groups	Mortality/Total	Morbidity (%)	Protective efficacy (%)
PBS i.m.	10/10	100	0
Blank Ag@SiO_2_-NPs i.m.	10/10	100	0
pVAX I-F (o) DNA i.m.	3/10	30	70
pFDNA-Ag@SiO_2_-NPs i.m.	2/10	20	80
pFDNA-Ag@SiO_2_-NPs i.n.	0/10	0	100
