# Supplementary material for: IgA response and protection following nasal vaccination of chickens with Newcastle disease virus DNA vaccine nanoencapsulated with Ag@SiO2 hollow nanoparticles
Source: Sci Rep. 2016 May 12;6:25720. doi: 10.1038/srep25720 (PMC4864420; doi:10.1038/srep25720)
Supplement: Supplementary Information [file srep25720-s1.pdf]

IgA response and protection following nasal vaccination of chickens with Newcastle disease virus DNA vaccine nanoencapsulated with $Ag@SiO_2$ hollow nanoparticles

Kai Zhao [1, 2, *], Guangyu Rong [2, 4], Yan Hao [2], Lu Yu [2, 4], Hong Kang [2], Xin Wang [2], Xiaohua Wang [2], Zheng Jin [3], Zhiyu Ren [3, *], Zejun Li [4, *]

[1] School of Biological Science and Technology, University of Jinan, Jinan 250022, China

[2] Key Laboratory of Microbiology, School of Life Science, Heilongjiang University, Harbin 150080, PR China;

[3] Key Laboratory of Functional Inorganic Material Chemistry, Ministry of Education, Laboratory of Physical Chemistry, Key Laboratory of Chemical Engineering Process & Technology for High-efficiency Conversion, School of Chemistry and Materials Science, Heilongjiang University, Harbin 150080, PR China;

[4] Department of Avian Infectious Disease, Shanghai Veterinary Research Institute, Chinese Academy of Agricultural Sciences, Shanghai 200241, PR China;

Running title: $Ag@SiO_2$ nanoparticles as an efficient vaccine delivery carrier

* Correspondence: Kai Zhao, Tel: +86 531 82767361, Fax: +86 531 89736818, E-mail: zybin395@126.com; Zhiyu Ren, Tel: +86 0 18946066093, Fax: +86 451 51997166, E-mail: zyren@hlju.edu.cn; Zejun Li, E-mail: lizejun@shvri.ac.cn

Supplementary Information

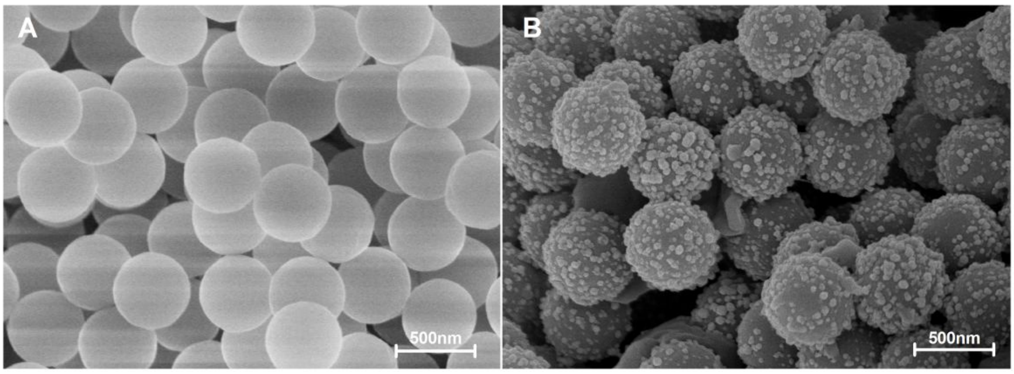

Supplementary Figure 1. Scanning electron microscopy micrograph of (A) PS microspheres and (B) PS@Ag.

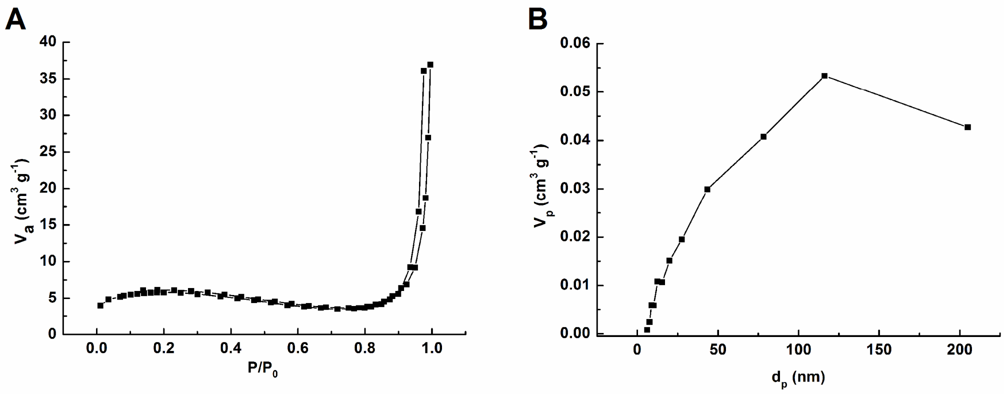

Supplementary Figure 2. (A) N_2 adsorption–desorption isotherms and (B) the corresponding BJH pore size distribution plots of samples Ag@SiO_2 hollow nanoparticles.

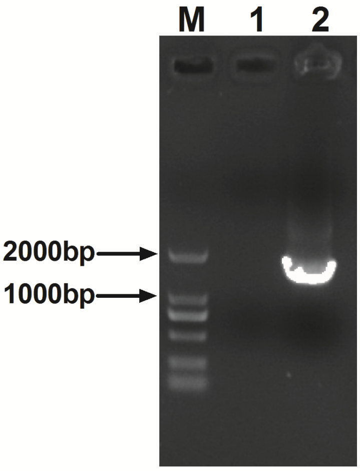

Supplementary Figure 3. The PCR amplification of F genes. Lane 1 shows F genes amplification sequences of the plasmid pVAXⅠ-F (o) DNA treated by DNase Ⅰ; Lane 2 shows F genes amplification sequences of the pFDNA-Ag@SiO_2-NPs treated by DNase Ⅰ; M: DNA Marker DL 2000.

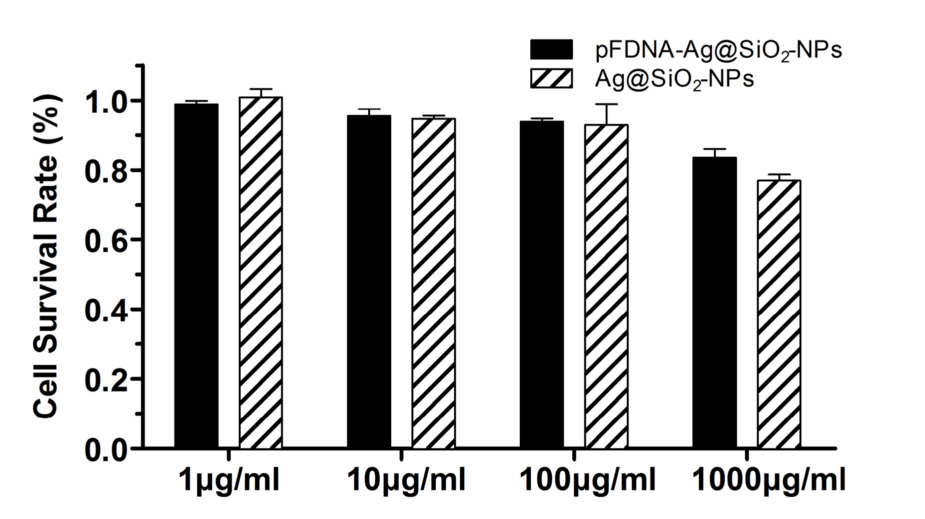

Supplementary Figure 4. *In vitro* cytotoxicity analysis of CEF cells survival ratio treated with different concentrations of pFDNA-Ag@SiO_2-NPs and Ag@SiO_2 hollow nanoparticles. Error bar represented means of three independent experiments.
